# Supplementary material for: Air-ground collaborative multi-source orbital integrated detection system: Combining 3D imaging and intrusion recognition
Source: PLoS One. 2025 Jul 7;20(7):e0326951. doi: 10.1371/journal.pone.0326951 (PMC12233268; doi:10.1371/journal.pone.0326951)
Supplement: Attachment — Submitted filename: Response to Reviewers.docx [file pone.0326951.s002.docx]

**Response to Reviewers**

**Manuscript ID:** PONE-D-25-14222

**Title:** Air-Ground Collaborative Multi-Source Orbital Integrated Detection System: Combining 3D Imaging and Intrusion Recognition

**Authors:** Mengyuan Yan, Xingyu Yang*, Wei Gao, Lifan Rong, Shengbo Li, Yuan Xiong

We sincerely thank the editors and reviewers for their constructive feedback, which has significantly improved the quality of our manuscript. Below, we provide detailed responses to each comment and specify the revisions made to the manuscript. All changes are highlighted in blue in the revised manuscript (Document 2).

**Response to Reviewer 1**

**Comment 1:** "You relied exclusively on Chinese sources, which are valuable, but there have also been relevant HORIZON projects in Europe, with numerous publications on this topic. In addition, some private companies have developed similar systems.“

**Response:** We appreciate this suggestion. In the revised manuscript , we have expanded the literature review to include European initiatives and commercial systems:

Added a discussion of the EU HORIZON 2020 SAFER-LC project, which utilizes millimeter-wave radar for foggy conditions.

Referenced PerceptRail's vision-LiDAR fusion system and compared its limitations (17.3% false-negative rate for overhead obstacles) with our system’s performance.

Cited recent European studies, including Smith et al. (2023) and European Union Agency for Railways (2023) .

These additions provide a global perspective on railway inspection technologies and highlight the novelty of our approach.

**Comment 2:** "It would be beneficial to provide more details about the software part. This is essential for the reproducibility of the results; if someone wanted to replicate your experiment, they should be able to do so based on your paper.“

**Response:** We have added comprehensive software implementation details:

ROS Framework: Described the use of ROS Melodic for sensor synchronization and real-time processing.

Data Synchronization: Specified GPS timestamp alignment with microsecond precision and custom ROS protocols .

Algorithm Implementation: Expanded the explanation of the improved LOAM-SLAM algorithm and NDT initialization.

These revisions appear in Sections "System Design" and "Dynamic Mapping and Registration" of the revised manuscript.

**Comment 3:** " In Table 3, you present the training times for the neural network. Please add information on dataset size and other relevant details."

**Response:** We have updated the Data Preparation section to include:

Dataset size: 25,000 images (15,000 field-collected + 10,000 from RailSem19).

Data augmentation methods: Spatial transformations (rotation, flipping), pixel-level adjustments (brightness, noise), and normalization.

These details ensure reproducibility and transparency.

**Comment 4:** "The conclusion is rather brief. I found more information in the abstract than in the conclusion. Strengthen the conclusion so that a reader who only reviews the abstract and conclusion can still find all the relevant insights."

**Response:** We have substantially expanded the Conclusions section:

Highlighted quantitative improvements: 1,200 pts/m² point cloud density, 4.3±0.4 mm registration error, and 25 FPS intrusion detection.

Emphasized real-world validation in foggy conditions (95% accuracy) and operational cost reduction (35%).

Outlined future directions: Integration of millimeter-wave radar and edge-cloud frameworks.

These revisions align the conclusion with the abstract’s depth while adding new technical and practical insights.

**Response to Reviewer 2**

**Comment 1**: "here are very few literature summaries of studies related to the study topic or similar topics. The comparison of the findings of the study with the findings of studies in the literature is almost nonexistent. "

**Response:**

We have strengthened the literature review and methodology:

Comparative Analysis: Benchmark our system against manual inspection, dedicated trains, and fixed monitoring systems.

Technical Enhancements:

Detailed multi-LiDAR calibration and motion distortion correction.

Explained the improved ICP algorithm with feature-weighted matching.

Performance Validation: Compared our YOLOv3-ResNet model with Faster R-CNN, YOLOv5s, and RetinaNet.

These revisions address the lack of technical and comparative clarity in the original manuscript.

**Comment 2:** " Providing more detailed information about the study method would facilitate the comprehensibility of the study." **Response:** We have added granular methodological details: Software Workflow:

Timestamp alignment using linear motion equations .

Depth map projection for cross-modal data fusion .

Open-Source Tools: Utilized PCL libraries for RGB fusion and CloudCompare for visualization.

These additions appear in Sections "Hardware Configuration" and "Colorization and Visualization."

**Conclusion**

We thank the reviewers for their rigorous evaluation. All suggested revisions have been incorporated to enhance the manuscript’s scientific rigor, technical clarity, and global relevance. The revised version demonstrates significant advancements in railway inspection through hardware innovation, algorithmic improvements, and robust validation.

Sincerely,

Mengyuan Yan, Xingyu Yang*, Wei Gao, Lifan Rong, Shengbo Li, Yuan Xiong

Shijiazhuang Tiedao University, China
